# Wine Consumption and Oral Cavity Cancer: Friend or Foe, Two Faces of Janus

**DOI:** 10.3390/molecules25112569

**Published:** 2020-05-31

**Authors:** Paula Silva, Norbert Latruffe, Giovanni de Gaetano

**Affiliations:** 1 Laboratory of Histology and Embryology, Institute of Biomedical Sciences Abel Salazar (ICBAS), University of Porto, Rua de Jorge Viterbo Ferreira nº228, 4050-313 Porto, Portugal; 2 BioPeroxIL laboratory, Université de Bourgogne, 6, Boulevard Gabriel, 21000 Dijon, France; Norbert.Latruffe@u-bourgogne.fr; 3 Department of Epidemiology and Prevention, IRCCS Istituto Neurologico Mediterraneo Neuromed, 86077 Pozzilli, Italy; giovanni.degaetano@moli-sani.org or

**Keywords:** wine, ethanol, acetaldehyde, oral cavity cancer, carcinogenesis, resveratrol

## Abstract

The health benefits of moderate wine consumption have been extensively studied during the last few decades. Some studies have demonstrated protective associations between moderate drinking and several diseases including oral cavity cancer (OCC). However, due to the various adverse effects related to ethanol content, the recommendation of moderate wine consumption has been controversial. The polyphenolic components of wine contribute to its beneficial effects with different biological pathways, including antioxidant, lipid regulating and anti-inflammatory effects. On the other hand, in the oral cavity, ethanol is oxidized to form acetaldehyde, a metabolite with genotoxic properties. This review is a critical compilation of both the beneficial and the detrimental effects of wine consumption on OCC.

## 1. Introduction

Oral cavity cancer (OCC) is a neoplastic condition characterized by the malignant transformation of the lips, oral cavity or oropharynx cells. In 2018, the worldwide estimate was 177,384 deaths and 354,864 new cases of OCC, which is the fourth most common cancer and the sixth most common cause of cancer deaths in low- and middle-income countries [1]. The consumption of alcoholic beverages has been pointed out as one key risk factor for OCC. The population-attributable risk of OCC for alcohol consumption alone is lower than 18% [2]. Epidemiological studies indicate that the risk associated with OCC increases when it is treated as an independent effect in people who consume ≥30 grams of ethanol per day [3,4,5,6,7,8,9,10,11,12]. The relative risk of cancers of the oral cavity and pharynx, esophagus and larynx are around five for an amount of around 50 g/day of ethanol [13]. These values are higher than the ones that define moderate consumption (up to one drink—equivalent to about 12 g of ethanol—per day in women and up to two in men, of all types of alcoholic beverages combined) [13]. Higher consumption, of more than three drinks per day, over a short period (a few years) has a higher risk of oral cancer than a lower intake over a longer period (many years) [14].

Wine is known for its large quantities of polyphenols, which have antioxidant properties that may counteract the potential pro-oxidant effect of ethanol. Numerous studies of animals and humans have shown that the bioavailability of phenolic compounds is low [15]. However, oral cavity tissues are in direct contact with wine and its compounds. The levels of salivary polyphenols peaked soon after red wine intake in healthy volunteers [16,17]. The effects of phenolic compounds in the oral cavity derive mainly from a reservoir adhering to oral mucosa rather than from systemic absorption. Therefore, it seems that the intra-oral actions of both ethanol and the phenolic portion of the wine overlap with the systemic ones, which makes OCC a peculiar type of disease to study.

In this review, we analyze the molecular mechanisms of ethanol-related carcinogenesis and phenolic-related preventive-carcinogenesis in the oral cavity and explore the possibility of a dual contrasting effect of these wine components in the development of OCC.

## 2. Wine as Oral Cavity Cancer-Enhancer

### 2.1. Formation and Accumulation of Acetaldehyde in Oral Cavity after Wine Ingestion

**Figure 1:**
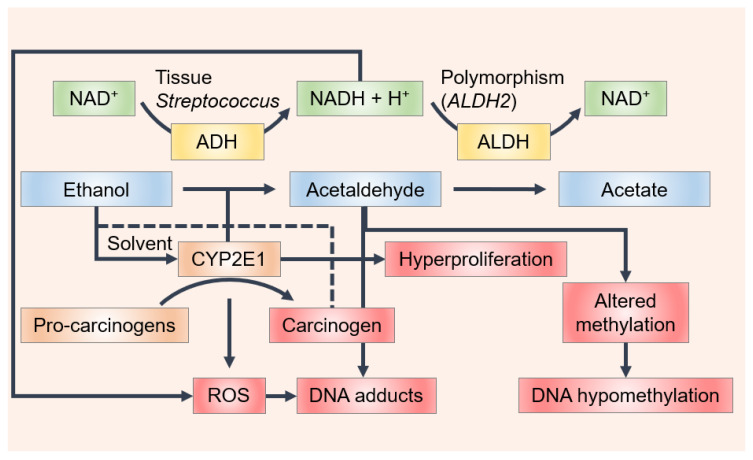
Scheme of the mechanisms by which ethanol may affect oral carcinogenesis. Ethanol is metabolized to form acetaldehyde by alcohol dehydrogenase (ADH) and cytochrome P450 2E1 (CYP2E1) in the oral cavity and is further oxidized to form acetate by acetaldehyde dehydrogenase (ALDH). ADH-mediated ethanol metabolism results in the generation of reducing equivalents in the form of reduced nicotinamide adenine dinucleotide (NADH) and acetaldehyde, whereas ethanol oxidation by CYP2E1 leads to the production of acetaldehyde but also to the generation of reactive oxygen species (ROS). Single nucleotide polymorphisms of ALDH2 cause the production and/or oxidation of acetaldehyde to vary between individuals. Increased CYP2E1 activity not only leads to an increased generation of ROS but also leads to an increased activation of various environmental agents such as the pro-carcinogens present in tobacco smoke. Ethanol may also act as a solvent for these carcinogens to enter the cell. Acetaldehyde can bind to DNA, forming stable adducts, and ROS results in lipid peroxidation products, such as 4-hydroxynonenal (4-HNE), which bind to DNA to form mutagenic adducts. During cancer promotion, ethanol and acetaldehyde alter methyl transfer, leading to DNA hypomethylation that could change the expression of oncogenes and tumor-suppressor genes. Finally, ethanol-associated immune suppression may facilitate tumor cell spreading.

Wine contains ethanol, which by itself is not a carcinogen; however, acetaldehyde, which is associated with wine consumption, is classified as “carcinogenic to humans” by the International Agency for Research on Cancer (IARC), based, in large part, on the elevated risk of oral and esophageal cancers in alcohol abusers [18,19]. The concentration of acetaldehyde varies among wine types (e.g., white, red, sparkling and fortified wines) as a result of the different winemaking conditions, particularly with the quantity of SO_2_ added to the medium. Therefore, different values appear in the literature. Acetaldehyde has been detected at concentration levels of 80 mg/L for white wines, 30 mg/L for red wines and 300 mg/L for sherries [20]. Jackowetz and Orduña [21] reported a final wine concentration of acetaldehyde of 25 mg/L in reds and 40 mg/L in white wines. Different values were found in another study, where acetaldehyde content was measured in a large collection of different alcoholic beverages (over 1500 samples), in which the amount found was 34 mg/L and 118 ± 120 mg/L in wine and in fortified wines, respectively [22]. In a study carried out to measure the acetaldehyde concentration in different beverages consumed in Italy, acetaldehyde concentrations of 55.8 mg/L in red, 67 mg/L in white, 81.7 mg/L in rosé and 123 mg/L in sparkling wine and champagne were found [23]. Linderborg et al. [24] found a lower concentration of acetaldehyde in wine samples (12.1 mg/L ± 10.4 mg/L). Recently, acetaldehyde levels ranging from 2.49 ± 0.34 to 29.27 ± 4.69 mg/L were found in Cabernet Sauvignon wines and this declined by close to 40% during aging under screw cap closures which admitted very little oxygen [25]. Despite the differences found among studies, wine contains acetaldehyde levels above the mutagenic limit (4.4 mg/L). Moreover, the IARC classification includes both acetaldehyde present in wine and acetaldehyde formed from ethanol via endogenous metabolism [22,24,26,27,28]. In fact, one of the key mechanisms in the oral formation of acetaldehyde is the metabolism of ethanol by the microbial flora of the oral mucosa [18,29,30]. Ethanol is oxidized by mucosal and microbial cells to form acetaldehyde by alcohol dehydrogenase (ADH), mainly alcohol dehydrogenase-1B (ADH1B) (Figure 1). Acetaldehyde is further metabolized by aldehyde dehydrogenase (ALDH, mostly by aldehyde dehydrogenase-2 (ALDH2)), yielding acetate, which is a less toxic and less harmful compound (Figure 1). Despite this process primarily occurring in the liver, the required enzymes are also expressed in the oral mucosa and gingiva. Oral microflora appears to be the main origin of acetaldehyde concentration in saliva. Some *Streptococcus* species have produced high quantities of acetaldehyde and showed significant ADH activity, suggesting that they may participate in metabolizing ethanol to form carcinogenic acetaldehyde in the oral cavity (Figure 1) [31]. As revealed by an in vitro characterization of the oral microbiome, both the *Neisseria* and *Candida* species are among the most potent microbial producers of acetaldehyde [32,33,34,35].

From that which has been reported above, it is clear that in order to evaluate the OCC risk of wine, it is important to measure the acetaldehyde content in saliva after wine ingestion. In vivo findings in humans have shown that acetaldehyde concentrations in saliva range between 0.793 mg/L and 4.41 mg/L after a dose of alcohol containing 0.5 g ethanol/kg body weight [17,36,37]. A study that was carried out to clarify the effects of alcohol beverage type on salivary and blood acetaldehyde and ethanol levels, after a moderate dose of alcoholic beverages in healthy Japanese volunteers, showed that the type of alcoholic beverage (13% ethanol Calvados, 13% ethanol shochu, 13% ethanol red wine and 5% ethanol beer) had no effect on the salivary acetaldehyde levels that were measured 30 min or more after the completion of drinking. However, the salivary acetaldehyde concentration after drinking red wine was significantly lower than that after drinking any of the other beverages [36].

Ethanol may also be metabolized to form acetaldehyde by the cytochrome P450 2E1 (CYP2E1) present in the keratinocytes of buccal mucosa (Figure 1) [38]. The increase in CYP2E1 activity is due to ethanol consumption and, consequently, the generation of reactive oxygen and reactive nitrogen species (ROS, RNS). Some studies suggest that the initiation of OCC results from DNA damage by ROS/RNS via the activation of proto-oncogenes and the inactivation of tumor suppressor genes. An accumulation of 8-nitroguanine, which is a potentially mutagenic DNA lesion, and 8-hydroxy-deoxyguanosine, one of the most frequent DNA base modifications associated with oxidative damage, has been found in the tissue of patients with oral lichen planus (OLP) [38,39], oral squamous cell carcinoma (OSCC) [38] and leucoplakia [40], though no immune-reactivity was observed in normal oral mucosa [38]. The formation of 8-nitroguanine and 8-oxodG may contribute to the development of oral cancer from OLP and leucoplakia [41]. It was also observed that inducible nitric oxide synthase dependent DNA damage may stimulate tumor protein p53 accumulation in OLP, leukoplakia and OSCC [41]. Increased levels of 4-hydroxy-2-nonenal and malondialdehyde, which result from the lipid peroxidation of cell membranes by ROS, have been reported in oral cancer and pre-cancer patients [42,43,44,45].

Ethanol may directly affect the oral mucosa since it can act as a solvent, removing some of mucosa lipid content, thereby making it considerably more permeable, which also facilitates the development of tumors on such exposed locations by the increased absorption of other carcinogenic substances [46,47,48].

### 2.2. Ethanol/Acetaldehyde Genotoxicity

**Figure 2:**
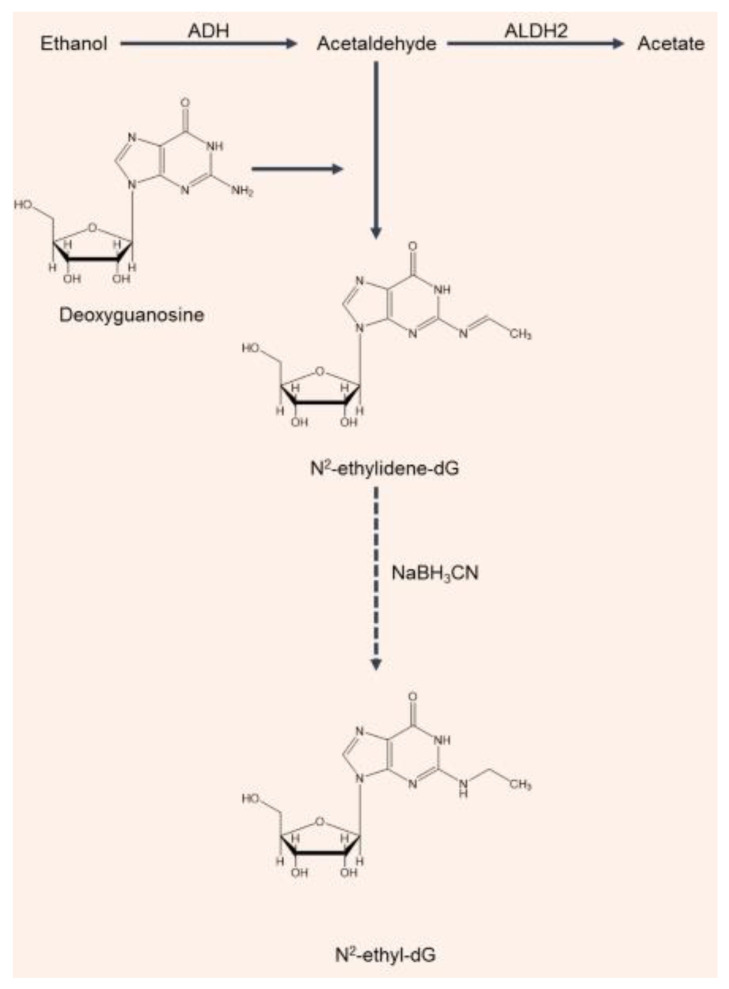
Formation of the N2-ethylidene-dG adduct and the N2-ethyl-dG adduct due to acetaldehyde production from ethanol. Acetaldehyde can interact with deoxyguanosine (dG) to form a Schiff base N2-ethylidene-dG. During the reduction step, the unstable N2-ethylidene-dG is expected to be converted to the stable N2-ethyl-dG.

Acetaldehyde’s genotoxicity is linked to its reactivity, forming DNA adducts and interfering with DNA synthesis and repair as well as binding to proteins, altering their structure and function. Mutagenic DNA adducts can be formed when acetaldehyde is present in concentrations equal to or higher than 6.30 mg/L [49,50]. The major acetaldehyde-derived DNA adduct in the human body is a Schiff base, N2-ethylidene-2′-deoxyguanosine (N2-ethylidene-dG) [51]. Since the N2-ethylidene-dG adduct is unstable in the single 2′-deoxynucleoside form, with a half-life of just 5 min, an analytical approach was developed for quantifying N2-ethyl-2′-deoxyguanosine (N2-ethyl-dG): this is a compound which is more stable and easier to detect than results from the reduction of N2-ethylidene-dG by sodium cyanoborohydride (NaBH_3_CN) (Figure 2) [52]. Therefore, for assessing the effects of alcohol consumption on DNA in studies of alcohol-related carcinogenicity, N2-ethyl-dG has been used as a biomarker (Figure 2). The detection of N2-ethyl-dG supported epidemiological studies showing a higher risk of oral and esophageal cancer in ALDH2-deficient individuals who drink chronically [53]. Balbo et al. [54] used N2-ethyl-dG to investigate, for the first time, the effects of alcohol consumption on the time course of DNA adduct production in the oral cavities of healthy volunteers. A clear dose–response relationship between the levels of N2-ethyl-dG produced and the amount of alcohol consumed was observed. The most interesting result of this bio-kinetic study was that the adduct levels returned to baseline values after 24 h. Since the half-life of N2-ethylidene-dG in DNA is 24 h at 37 °C, the elimination of adducts can be explained by either DNA repair or cell turnover [54,55]. It is possible that the nucleotide excision repair mechanism could remove the lesion, since neither base deletion repair nor direct repair have been shown to be able to remove N2-ethyl-dG (used as a substitute for N2-ethylidene-dG). The other possibility is that the return of the adduct levels to baseline values reflects changes in the cell population that is being sampled. Cells in the basal layer of the epithelium appropriately undergo mitosis to provide cell renewal. As these cells differentiate, they are pushed toward the surface by new cells in the basal layer. Therefore, the cells sampled at the 24 h time point would have been in a different epithelial layer relative to the surface during the alcohol drinking and immediately afterwards, when salivary acetaldehyde levels would be at their highest [54,55]. The condensation of two molecules of acetaldehyde may also produce a reactive electrophile, croton-aldehyde, which can also form a Schiff base on the same amino group of deoxyguanosine (dG), which results in the formation of other adducts the croton-aldehyde-derived propano-dG ones. Under the in vitro conditions that were investigated, these adducts proved to be very unstable. Further investigation is needed to clarify the biological significance of these adducts [56].

### 2.3. Ethanol/Acetaldehyde and Pre-Cancerous Lesions

**Figure 3:**
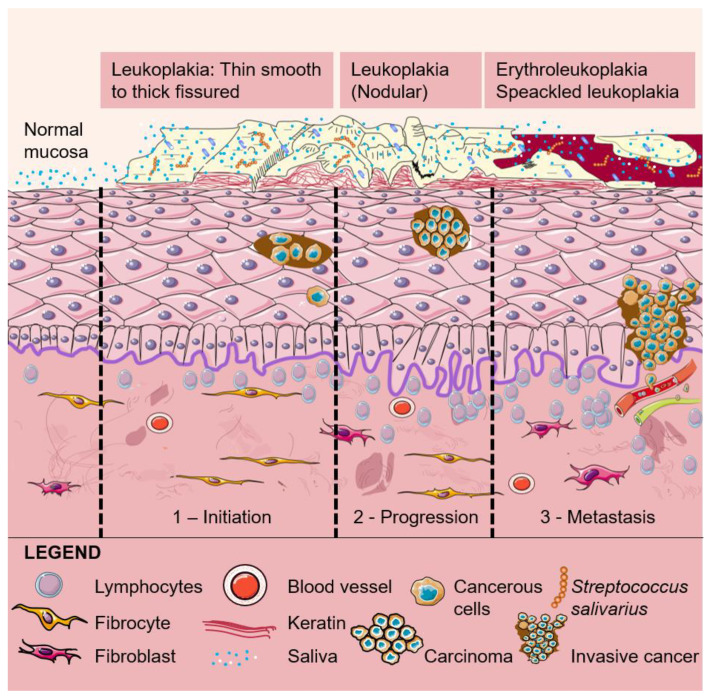
The various clinical appearances of leukoplakia with expected underlying microscopic changes are shown. Leukoplakia is a pre-malignant, pre-cancerous or potentially malignant lesion or condition, which means that there is an increased risk of future malignant transformation into a squamous cell carcinoma either at the site of the leukoplakia or elsewhere in the oral cavity. Lesions become progressively more “severe” toward the right, culminating in erythroleukoplakia, which most frequently demonstrates severe epithelial dysplasia and carcinoma in situ when studied histologically. It should be emphasized that the scheme does not necessarily represent a chronological change, but rather it shows the potential presentations of leukoplakia. Homogeneous leukoplakia is a uniform, flat, thin and white plaque, with or without fissuring and with a gradual increase of hyperkeratosis and acanthosis. Leukoplakia can also be non-homogeneous, being nodular or flat with a mixed white and red discoloration (“erythroleukoplakia”). The histopathologic features of leukoplakia may vary from hyperkeratosis with or without epithelial dysplasia to various degrees of epithelial dysplasia, carcinoma in situ and even invasive squamous cell carcinoma.

Acetaldehyde also damages oral mucosa, which promotes the stimulation of cell regeneration. DNA mutation may result from the spreading out of the proliferative cell compartment and hyper-regeneration. The various alterations in DNA can progress from a normal oral epithelial cell to a pre-malignant or a potentially malignant oral epithelial cell that is characterized by the ability to proliferate in a non-controlled mode. Genetic alterations may then cause the development of pre-cancerous lesions, which develop in the form of benign or malignant tumors. Pre-cancerous lesions can be in the form of leukoplakia, erythroplakia, erythroleukoplakia (Figure 3) or oral sub-mucous fibrosis, and all these can potentially give rise to a primary tumor in the oral cavity [56,57,58,59,60]. OCC involves changes in the mucosal layers that most probably occur in the entire epithelial surface of the oral cavity and are followed by the invasion of tumor cells [61]. Changes in over approximately 100 genes have been involved in OCC, the overexpression of oncogenes and/or the silencing of tumor suppressor genes being the focus of the scientific community [62].

A case–control study in Kenya revealed a weak to moderate association between wine intake and oral leukoplakia [63]. No relationship was found in a case–control study investigating the role of alcohol consumption in the development of oral leukoplakia in Southern Taiwan. Subjects who had drunk a bottle or more of an alcoholic beverage per month for at least one year did not develop oral leukoplakia [64]. According to Petti et al. [65], regular intake of a moderate quantity of wine could reduce the risk of oral leukoplakia. Consumption of beer and hard liquor, but not wine, is more strongly associated with oral cancer than oral epithelial dysplasia [66]. Jaber et al. [67] carried out a study that aimed to provide an assessment of the importance of tobacco and alcohol consumption in the development of oral epithelial dysplasia in a large group of European patients. These authors found no relationship between the degree of wine consumption and risk of oral epithelial dysplasia. However, increased risk of oral epithelial dysplasia was associated with the consumption of fortified wines [67]. A retrospective case–control study showed no overall increased risk from wine and other alcoholic beverages of oral dysplasia. However, the proportion of subjects who drank spirits was significantly higher among cases than controls [68]. Morse et al. [69] found a two-fold increase in the risk of oral epithelial dysplasia associated with drinking seven or more drinks of beer and hard liquor per week but no excess risk with drinking an equivalent amount of wine. A study in Puerto Rico also suggests that any type of alcoholic beverage consumption, including wine, is positively associated with an increased risk of potentially malignant oral disorders [70]. Conflicting evidence exists to support alcohol’s role in the development of pre-cancerous lesions but apparently wine has little to no effect on their development. In general, these results corroborate the ones obtained by Purdue et al. [71], who found that among wine-only drinkers, the odds ratio for moderate levels of consumption frequency approached null. According to their study, only individuals with higher wine consumption levels were comparable to drinkers of other beverage types.

In summary, it seems that ethanol may act as an OCC promoter by multiple pathways. However, important questions remain to be answered about the mechanistic and dynamic bases of this relationship.

## 3. Wine as Oral Cavity Cancer-Preventer

Grapes contain phenolic compounds: these are highly specific metabolites that are important in plant regulatory mechanisms and play an important role in the response and resistance of plants to infection by pathogenic microorganisms. Phenolic compounds also directly contribute to the sensory properties, such as color, astringency, bitterness and roughness, of wine. They are involved in redox reactions, protein interactions and wine-aging processes [72]. The primary constituents of the phenolic compounds are flavonoids and non-flavonoids. Flavonoids make up approximately 85% of the total phenolic content of red wine but less than 20% of that of white wine [73]. Phenolic compounds have important effects on human physiology and are considered to have beneficial effects in relation to cancer and diabetes, microbial, inflammatory, neurodegenerative and kidney diseases and aging [74]. Herein, we will review the literature related to the chemoprevention potential of wine polyphenols, i.e., their potential for controlling the transformation of pre-malignant or potentially malignant lesions into invasive OCC.

### 3.1. In Vitro Studies

Wine contains the same flavanol derivatives as green tea, namely catechins; the latter have been extensively studied for their chemo-preventive potential, showing efficacy against multiple cancers including OCC [75,76]. Therefore, it is reasonable to suppose that wine flavanols could have similar OCC protective effects. Oral and head and neck cancer cells exposed to green tea and epigallocatechin-3-gallate (EGCG), respectively, lead to a decrease in the expression of the phosphorylated epidermal growth factor receptor (EGFR), suggesting that catechins are potential cancer chemo-therapeutic or chemo-preventive agents [77,78]. In vitro, tea catechins promote a decrease in the proliferation of different human head and neck squamous cell carcinoma (HNSCC) cell lines [77,78,79,80,81,82]. Li et al. [82] found that EGCG affects the proliferation, apoptosis, migration and invasion of tongue squamous cell carcinoma cells through the Hippo-TAZ signaling pathway. Polyphenols extracted from green tea have a synergistic beneficial effect with lactoferrin on oral carcinoma cells’ cytotoxicity and apoptosis. Moreover, polyphenols alone induce G0/G1 cell-cycle arrest and apoptosis [83]. EGCG also induces the G1 phase arrest of human OSCC cells [81]. Activation of the p53 tumor suppressor gene by green tea polyphenols could explain the induction of cell cycle arrest and apoptosis [84]. Treatment with EGCG increases caspase-3 and -7 activities and the percentage of apoptotic cells [81]. In addition, it was observed that EGCG induces cell apoptosis and autophagy and inhibits multi-drug resistance gene (MDR1) expression in oral cancer cells [85]. The in vitro effects of EGCG on oral cancer cells include three main phases: (i) inhibition of cell proliferation via apoptosis induction and cell cycle arrest; (ii) modulation of transcription factors, namely nuclear factor kappa-light-chain-enhancer (NF-κB) and activator protein; and (iii) reduction of cell migration and invasion by decreasing the production of matrix metallo-proteinases (MMPs) [86,87].

Quercetin is an efficient anti-cancer agent as evidenced by an EGFR decrease in EGFR-overexpressing HNSCC [88]. An in vitro study with human OSCC cells suggested that quercetin chemo-preventive mechanisms start by inducing a stress response, resulting in cell necrosis. Then, the surviving cells die by apoptosis after prolonged exposure to quercetin, presumably mediated by the inhibition of thymidylate synthase protein, a key S-phase enzyme [89].

The combination of quercetin with chemo-therapeutic drugs not only induces apoptosis but also decreases the cells’ resistance to the chemo-therapeutic medication [90,91]. The bio-pharmacological effects of quercetin on cell growth and invasion/migration inhibition involve cellular and molecular mechanisms, mainly via cell cycle arrest accompanied by mitochondria-mediated apoptosis. The caspase-3-dependent apoptosis of OSCC cells is one of the mechanisms that has been proposed to explain the anti-OCC properties of quercetin [92].

Quercetin efficiently inhibits the cellular migration and invasion of the HNSCC cell lines, HSC-3 and FaDu, and human oral cancer cells (SAS) via suppression of the MMP-2 and MMP-9 activation [88,93]. MMPs inhibition occurs via the down-regulation of protein kinase C and the blocking of mitogen activated protein kinases (MAPK) and phosphatidylinositide-3 kinases (PI3K) signaling pathways and both cyclo-oxygenase-2 (COX-2) and NF-κB [93]. Moreover, quercetin affects the ratio of anti-/pro-apoptotic proteins in SAS cell lines, which may lead to the dysfunction of mitochondria followed by the release of cytochrome c (cyto c), apoptosis-inducing factors and endonuclease G from mitochondria, inducing cell-destruction by triggering apoptosis [94].

Quercetin treatment enhances microRNA-16 (miR-16) expression and inhibits homeobox A10 (HOXA10) levels. The overexpression of miR-16 blocks cell viability, migration and invasion by targeting HOXA10, and its knockdown reverses the quercetin-mediated progression of oral cancer cells [95].

Several lines of evidence both in vitro and in vivo support the notion that quercetin is a potential therapeutic agent for a subset of human OSCC involving the activation of fork-head box O (FOXO1). In fact, quercetin suppresses cancer cell growth and promotes phase G2 cell cycle arrest and apoptosis in EGFR-overexpressing HSC-3 and TW206 cells, thus inducing the activation of FOXO1, the knockdown of which attenuates the quercetin induction of p21 and Fas ligand (FasL) expression [96]. From the above, it can be concluded that quercetin exerts chemo-preventive effects on the oral keratinocytes, and after a tumor has formed, quercetin could continue to have beneficial anti-tumor effects at higher doses by exerting cytotoxic effects.

Anthocyanins are flavonoids found mainly in grape skin and are responsible for the bluish-red color of the skin of red grapes and, therefore, for the color of red wine. Grape seed proanthocyanidins (GSPs) reduce cell viability and induce cell death in a dose- and time-dependent manner in human HNSCC cell lines from different sub-sites such as the oral cavity (SCC1), larynx (SCC5), tongue (OSC19) and pharynx (FaDu). GSPs reduce the expression of EGFR in those cell lines. Moreover, these anthocyanins increase the apoptosis of SCC1 and OSC19 cells with the induction of Bax (Bcl-2-associated X protein), reduction of the expression of Bcl-2 and the activation of caspase-3 [97]. GSPs inhibit the proliferation, migration and invasion of tongue squamous cell carcinoma cells (Tca8113) through suppression of the Akt/NF-κB signaling pathway [98].

Blueberries, a rich source of anthocyanins, and malvidin inhibit STAT-3 (signal transducers and activators of transcription-3), which prevents the proliferation and induces the apoptosis of oral cancer cells in vitro, a result further confirmed in vivo. Blueberry and malvidin suppress STAT-3 phosphorylation, block the nuclear translocation of the active dimer and prevent the transactivation of the STAT3 target genes that play crucial roles in cell proliferation and apoptosis [99]. Anthocyanins from the wild blueberries of Inner Mongolia suppress the growth of the oral cancer cell line KB in a dose-dependent manner as well as induce G2/M cell cycle arrest and apoptosis of the cells. Anthocyanin treatment increases the expression of caspase-9 and cyto c. Anthocyanins can also down-regulate the methylation of tumor protein p53 [100]. In a different study, it was observed that besides blueberry, cranberry, blackberry, black raspberry, red raspberry and strawberry extracts also inhibit the proliferation of human oral cancer cell lines [101,102]. The result for black raspberry was observed in another study in which extracts of this fruit inhibited the growth of oral pre-malignant and malignant cells by targeting cell cycle regulatory proteins [103]. Isolated cell lines from human OSCC tumors were used to investigate the effects of a freeze-dried black raspberry ethanol extract on cellular growth [104]. As in the other studies, black raspberry extracts suppressed cell proliferation without perturbing viability, inhibited the translation of the complete angiogenic cytokine vascular endothelial growth factor (VEGF), suppressed nitric oxide synthase activity and induced both apoptosis and terminal differentiation [104].

Crude extracts of strawberry and pure anthocyanins, namely cyanidin-3-*O*-glucoside, pelargonidin and pelargonidin-3-*O*-rutinoside, inhibit the proliferation of KB and CAL27 human oral cancer cells, which has been associated with an antioxidant mechanism of action [105]. In human oral CAL 27 cells, it has also been observed that anthocyanins from a species of black rice could decrease cells’ metastasis by the reduction of MMP-2, MMP-9 and NF-κB p65 expression through the suppression of the PI3K/Akt pathway and the inhibition of NF-κB levels [106]. Recently, it was shown that anthocyanin promotes the death of OSCC cells through the activation of pyroptosis [107].

The mechanism of action of anthocyanins seems to involve their ability to modulate epithelial cell growth and quench ROS, which is achieved because anthocyanins affect intracellular signaling and gene expression [108]. In fact, the anti-mutagenic and anti-carcinogenic activities of anthocyanins are generally ascribed to their antioxidant properties as conveyed by their phenolic structure. They may play an important role in the anti-cancer effects in OCC and are worthy of further investigation.

Resveratrol is a stilbene and the major non-flavonoid found in red wines, and it can modulate the signal transduction pathways that control cell division and growth, apoptosis, inflammation, angiogenesis and metastasis [109,110]. Its anti-cancer properties have been shown on various types of cancer cells including those of HNSCC origin [110,111]. Resveratrol’s anti-cancer effects are related to the inhibition of the proliferation of different oral cancer cells through the induction of apoptosis [112,113]. Moreover, resveratrol has considerable efficacy against the growth and proliferation of HNSCC through its selective induction of DNA damage and apoptosis, independently of Smad4 status, the mutation/absence of which is one of the primary causes of failed cellular DNA repair machinery in HNSCC [114].

Another study aimed to find potential compounds for the treatment of OCC, based on a large scale of reliable compound- and bioactivity-databases which showed that resveratrol is a natural product with a high potential to treat OCC. Resveratrol inhibits matrix MMP-9 expression and metastasis in oral cancer cells by down-regulating the signaling pathways of c-Jun N-terminal kinase1/2 and extra-cellular signal-regulated kinase1/2 signals, thus exerting beneficial effects in chemo-prevention [115]. Concentrations of 100 μM resveratrol decrease the adhesion, migration and invasion of OSCC cells (KB) [116] and of human oral cancer cell lines (SCC-9) [115]. Cell migration induced by 12-*O*-tetradecanoylphorbol-13-acetate (TPA) is also inhibited by resveratrol, which reduces the expression of MMP-9 and blocks the extra-cellular signal-regulated kinase (ERK) and JNK-MAPK (c-Jun N-terminal protein kinase family of mitogen-activated protein kinases) pathways. The reduction of MMP-9 activity by resveratrol is related to the suppression of the phosphorylation of ERK and JNK induced by TPA [115]. Using the oral cancer cell line SAS, it was observed that resveratrol induces apoptosis through nuclear factor-erythroid 2-related factor 2, heme oxygenase 1, tumor protein p53 and Bax signaling pathways [117].

The exposition of an OSCC cell line to a combination of resveratrol and doxorubicin loaded in liposomal nanoparticles exerts apoptosis-inducing effects by controlling the cell cycle and downstream apoptosis-inducing proteins such as caspase-3 and ribose polymerase-1 [118]. Their data indicate that the drug-loaded nanoparticle exerted apoptosis-inducing effects by controlling the cell cycle and downstream apoptosis by inducing proteins such as caspase-3 and poly (ADP-ribose) polymerase 1.

Nano-diamino-tetrac (NDAT) inhibits programmed death-ligand 1 (PD-L1) expression which is essential for proliferation in oral cancer cells. Recently, it was shown that a combined treatment of resveratrol and NDAT is more effective in reducing programmed death-ligand 1 expression and anti-proliferation as compared with resveratrol treatment alone in two oral cancer cell lines [119]. Thyroxine is an enhancer of the proliferation and progression of oral cancer cells by the down-regulation of apoptotic factor BAD (B-cell lymphoma 2 (Bcl-2)-associated agonist of cell death) and up-regulation of PD-L1. Resveratrol inhibits the function of thyroxine so that resveratrol supplementation enhances the expression of BAD and inhibits PD-1 to suppress oral cancer cells [120]. Chen et al [121] found that blocking expressions of inflammatory genes in oral cancer cells makes resveratrol an attractive agent that could possibly be employed in combination with other anti-STAT3 drugs.

Hayashi et al. [122] found that the overexpression of tripartite motif family-like 2 (TRIML2) contributes to tumor growth at the G1 phase as seen by cell cycle analysis, which results in insufficient control by the down-regulation of p21^Cip1^ expression. The authors also observed that resveratrol caused the up-regulation of p21^Cip1^ through the TRIML2 expression. Therefore, the authors concluded that the expression status of TRIML2 might be an indicator of OSCC progression and resveratrol may be a potential new therapeutic drug for oral cancer therapy via TRIML2 [122].

The combination of 50 µM resveratrol with 10, 25 and 50 µM of quercetin resulted in a significant inhibitory effect on cell growth and DNA synthesis [123]. Resveratrol is the major compound of *Polygonum cuspidatum* (PCE), which reduces human oral cancer cells’ viability in a concentration- and time-dependent mode PCE treatment induced autophagic and apoptotic cell death. PCE also stimulated caspase-9 and -3. These findings also suggest that resveratrol may be potentially efficacious for the treatment of cisplatin-resistant human oral cancer [124]. Pinostilbene hydrate, a methylated derivative of resveratrol, inhibits the migration and invasion ability, reducing the protein activity and expression of matrix MMP-2 in three oral cancer cell lines (SCC-9, SAS and HSC) by down-regulating the p38/ERK1/2 pathway, and it might be a promising agent for preventing OSCC cell metastasis [125].

### 3.2. In Vivo Studies

The chemo-preventive activity of grape skin extracts in oral carcinogenesis was evaluated in 4-nitroquinoline 1-oxide (4-NQO)-induced rats. After 12 weeks of treatment, a significant reduction in epithelial dysplasia was observed. Moreover, 8-hydroxy-2′–deoxyguanosine and ki-67 immuno-expression was reduced in animals treated with grape skin extracts. A Western blot analysis showed a significant decrease in p-NFκBp50 and myeloid differentiation primary response 88 protein expression in the groups treated with grape skin extracts. The authors concluded that grape skin extracts displayed chemo-preventive activity in oral carcinogenesis assays, as depicted by its antioxidant, anti-proliferative and anti-inflammatory properties [126].

Green tea polyphenols are also able to mitigate OCC in vivo. In 4-NQO-induced rats, green tea polyphenols decreased the levels of glutathione reductase and total thiols while increasing the levels of glutathione oxidase and conjugated dienes and increasing γ-glutamyl transferase activity. Supplementation with green tea polyphenols also reduced the activity of γ-glutamyl transferase, a tumor growth marker [127]. In a xenograft experiment on mice, EGCG treatment resulted in a 45.2% reduction in tumor size without a loss of body weight [81].

The chemo-prevention potential of quercetin has also been tested in vivo. Quercetin reduced tumor incidence and induced apoptosis through the modulation of NF-ĸB signaling and its target genes Bcl-2 and Bax in the DMBA (7,12-dimethylbenz(a)anthracene)-induced carcinogenesis hamster model [128].

In DMBA-induced hamster cheek pouch tumors, the dietary administration of freeze-dried black raspberries at a concentration of 5% of the diet inhibited the incidence, total number, multiplicity and size of tumors [129]. The environmental pollutant and tobacco smoke constituent dibenzo[def,p]chrysene (DBP) was used to induce OSCC in mice and to explore the effects of 5% dietary black raspberry. A reduction in the levels of DBP-DNA adducts in the mouse oral cavity with a comparable effect to those of its constituents was observed [130].

The effect of dietary GSPs was assessed on the in vivo tumor xenograft growth of SCC1 cells using athymic nude mice; these anthocyanins showed identical chemo-therapeutic efficacy to that which was observed in vitro in the same study, as mentioned above. This efficiency was found to be associated with the: (i) control of cell cycle regulation and (ii) induction of the apoptotic cell death of tumor cells, as indicated by the analysis of the proteins of the Bcl-2 family, TUNEL-positive and activated caspase-3-positive cells [97].

Resveratrol locally applied and complexed with 2-Hydroxypropyl-beta-cyclodextrin (HPβCD) (cream and mouthwash) in DMBA-induced OSCC in Syrian hamster cheek pouches prevents oral pre-neoplastic lesions and OSCC appearance and growth. HPβCD-formulations (mainly mouthwash) show the best chemo-preventive effects in terms of lesions’ prevalence, multiplicity, dimension and histological signs of malignancy [131]. Recently, an in vivo study was carried out using loaded GE11-conjugated liposomes (RSV-GL) and it was found that RSV-GL exhibited a two-fold decrease in tumor volume compared with the free resveratrol and a three-fold decrease in volume compared with the control [132].

### 3.3. Human Studies

There are few human studies, most of which have been conducted with green tea polyphenols. In a phase II clinical trial, patients with high-risk oral pre-malignant lesions receiving 500–1000 mg/m^2^ of green tea extract for 12 weeks exhibited reduced VEGF levels, which are angiogenic stimuli for tumors [133]. A double-blind intervention trial in patients with pre-cancerous lesions of the oral mucosa (leukoplakia) found that a treatment regimen of green and black tea polyphenols (3 g/day orally and a 10% ointment applied to lesions three times daily) resulted in lower numbers of micronucleated cells from oral lesions, normal oral mucosa and peripheral blood lymphocytes, thus providing some direct evidence for the protective effects of tea on OCC [134]. In patients with oral field cancerization, at a high risk for developing recurrent oral pre-cancerous and cancer lesions, EGCG was administered in a form of mouthwash for seven days and a decrease was found in the expression levels of some oral carcinogenesis biomarkers [135].

A clinical study was conducted to assess the effects of topical application of 10% freeze-dried black raspberry gel on oral intraepithelial neoplasia. The results showed histologic regression in a subset of patients and a reduction in the loss of heterozygosity at tumor suppressor gene-associated loci [136]. The berry gel application uniformly suppressed genes associated with RNA processing, growth factor recycling and the inhibition of apoptosis and suppression of epithelial COX-2 levels [137]. OSCC patients who were treated with black raspberries showed an enhanced expression of pro4-survival genes, such as EGFR, and a reduction in other pro-inflammatory genes, such as NF-kB1 and prostaglandin-endoperoxide synthase 2 [138].

Moreover, adherence to a Mediterranean diet based on ingredients of polyunsaturated fatty acid, polyphenols from olive oil and polyphenols from grapes, including the ones present in wine, decreased the risk of developing head and neck cancer [139].

## 4. Conclusions

At the experimental level, some studies were carried out to explore ethanol’s carcinogenic mechanisms whereas others analyzed the phenolic protective mechanisms. In the former group, the in vivo bio-kinetic studies were mainly focused on the analysis of salivary acetaldehyde. In contrast, the chemo-preventive/therapeutic properties of phenolic compounds against oral carcinogenesis were mainly studied using in vitro and in vivo test systems. Acetaldehyde resulting from wine intake damages oral mucosa, which promotes the stimulation of cell regeneration. The various alterations in DNA can result in the development of a pre-malignant or a potentially malignant oral epithelial cell characterized by the ability to proliferate in a non-controlled mode. In fact, acetaldehyde leads to the overexpression of oncogenes and/or the silencing of tumor suppressor genes. On other hand, several studies showed that polyphenols activate the p53 tumor suppressor gene. This could explain the induction of cell cycle arrest and apoptosis by polyphenols that was reported in some studies. Acetaldehyde’s genotoxicity also results in the formation of DNA adducts, which can also be reduced by polyphenols, as observed with black raspberry administration in vivo. On the other hand, polyphenols are potent antioxidants and, therefore, they counteract ROS/RNS generation due to an increase in CYP2E1 activity as promoted by ethanol consumption. Likely, the phenolic compounds from wine mitigate the deleterious effects of ethanol, decreasing the risk of OCC. Although all these studies have yielded important data for understanding the mechanisms of action of either ethanol or phenolic compounds on either normal or tumor keratinocyte cells from the oral cavity, much remains to be studied. More adequately powered, randomized, placebo-controlled human studies, as well as experimental animal models, are required for a better understanding of the effect(s) of wine, particularly when consumed regularly in moderate doses, on oral cells.

In conclusion, this area warrants further investigation as a new way of thinking, which is to assess the wine-specific intake risk while considering the additive/synergistic or contrasting effects of its different compounds.
